# Quantile index predictors using R package hyper.gam

**DOI:** 10.1093/bioinformatics/btaf430

**Published:** 2025-07-30

**Authors:** Tingting Zhan, Misung Yi, Inna Chervoneva

**Affiliations:** Division of Biostatistics & Bioinformatics, Department of Pharmacology, Physiology & Cancer Biology, Sidney Kimmel Medical College, Thomas Jefferson University, Philadelphia, PA 19107, United States; Department of Statistics & Data Science, College of Software and Convergence, Dankook University, Yongin-si, Gyeonggi-do, 16890, Korea; Division of Biostatistics & Bioinformatics, Department of Pharmacology, Physiology & Cancer Biology, Sidney Kimmel Medical College, Thomas Jefferson University, Philadelphia, PA 19107, United States

## Abstract

**Motivation:**

Evaluation of single-cell protein expression from immunohistochemistry images is used increasingly in biomedical research. Many proteins are used solely for phenotyping cells in the tumor microenvironment. Other proteins with meaningfully quantitative expression levels provide so-called functional protein biomarkers. There is still a limited number of methods and software tools available for utilizing the entire distributions of single-cell expression levels.

**Results:**

We present the R package hyper.gam, providing a supervised learning framework for deriving biomarkers based on single-cell distribution quantiles. The single-cell data are first converted into sample quantile functions, which are then used as predictors in scalar-on-function regression models to estimate the integrand surface. The estimated integrand surface defines the quantile index predictors based on the single-cell expression levels in a new test set. The package features a user-friendly interface and visual tools enabling exploration of the estimated integrand surfaces. Our tools are motivated by the need for biomarkers, taking into account heterogeneous protein expression levels in a tissue, but they can be applied to other types of single-cell data.

**Availability and implementation:**

R package hyper.gam and vignette are available at https://CRAN.R-project.org/package=hyper.gam and https://CRAN.R-project.org/package=hyper.gam/vignettes/applications.html.

## Introduction

Novel biomarkers are increasingly important for optimizing individualized cancer treatment strategy. Multiplex immuno-fluorescence immunohistochemistry (mIF-IHC) and advanced image analysis platforms enable measurement of protein expression in each single tumor cell, but some kind of average expression is usually considered as a potential biomarker. Meanwhile, intra-tumoral heterogeneity of protein expression in cancer cells is well-documented (e.g.Malinowsky *et al.* 2012, Qian *et al.* 2017) and may provide additional prognostic value (Supernat *et al.* 2014, Jacquemin *et al.* 2022).

Single-cell mIF-IHC data usually include coordinates of the cell centroids and cell signal intensity (CSI) of the protein expression and may be analyzed using spatial statistics approaches (e.g.Yuan, 2016, Wilson *et al.* 2021). Spatial analysis is especially informative for quantifying interaction between cancer and immune cells in the tumor immune microenvironment (e.g.Creed *et al.* 2021, Vu *et al.* 2022, Patrick *et al.* 2023). A spatial distribution may not be as relevant for quantification of heterogeneity for proteins that serve as functional biomarkers (Wrobel *et al.* 2023) with quantitative expression levels. The spatial approaches are computationally intense and often have complicated interpretations. Also, virtually all spatial metrics are based on the assumption of stationarity, which means that spatial localization patterns are similar in different regions of the tumor tissue. This assumption is generally not appropriate for the whole tumor tissues that always exhibit spatial heterogeneity of the protein expression of interest (e.g. Masotti *et al.* 2023).

Recently proposed approach for the *distributional* analysis of single-cell mIF-IHC data (Yi *et al.* 2023a) allows capturing intra-tumoral heterogeneity of protein expression by representing the entire distribution of CSIs in a tissue by the empirical/sample quantile function. The quantile function is the inverse of the cumulative distribution function. It is defined on the interval [0, 1] which facilitates the application of the functional data analysis methods. It is well-known that in case of independence between CSI levels and spatial localization of the cells, it is sufficient to consider marginal distribution of CSI without loss of information (e.g. Baddeley, 2013). Even if independence is not the case, considering CSI distributions without spatial localization of the cells may provide similar prognostic value. For example, Ki-67 expression was considered as a marker based on the spatial marked point pattern in Chervoneva *et al.* (2021) and based on quantiles of marginal CSIs distribution in Yi *et al.* (2023b), and results for predicting progression-free survival in breast cancer were very similar with and without considering spatial distribution of Ki-67 CSIs. Additional consideration is the much higher computational efficiency of non-spatial analysis approaches, which becomes more important for large volumes of single-cell mIF-IHC data.

In this article, we describe how to derive the Quantile Index (QI) predictors (Yi *et al.* 2023a, 2025) using the R package hyper.gam. The QI predictors are based on the empirical quantile functions of CSIs distributions in each biological sample. Prior to analysis, the R package groupedHyperframe is used to convert the CSI data into an R object of the groupedHyperframe class. In the first step of analysis, the single-cell data are converted into the sample quantiles of CSIs distributions on a pre-specified grid of probabilities. The sample quantile functions may have to be aggregated if more than two levels of clustering are present in the CSI data. In the second step, a suitable scalar-on-function model (Reiss *et al.* 2017) is fitted to a training data set with aggregated sample quantile functions as a predictor and a scalar outcome of interest. In the third step, the estimated integrand surface of the scalar-on-function regression model is used to compute a scalar quantile index based on the sample quantile function for each subject in the test dataset. The resulting quantile index can be evaluated as a predictor of outcome of the interest in the test data.

## Functionality and analysis

### Data structure and processing

**Figure 1.**
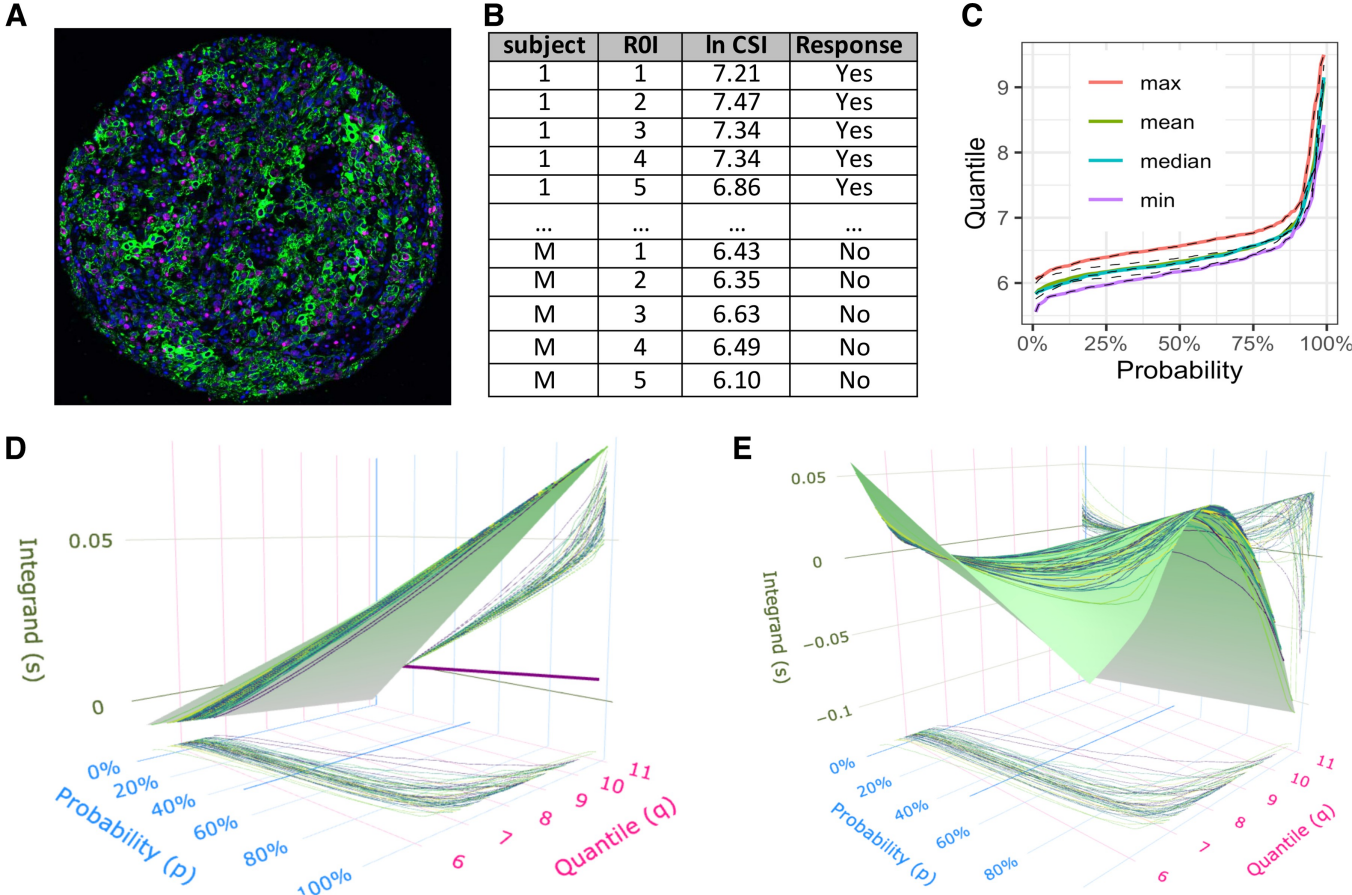
(A) Example of an mIF-IHC image of a tissue core stained for Ki-67 protein (pink) as well as DAPI (blue) and cytokeratin (green); (B) Clustered structure of the mIF-IHC CSI data used for deriving quantile biomarkers; (C) Example of ROI-specific quantile functions for one tissue (dashed black lines) and tissue-specific functions (colored solid lines) aggregated using point-wise max, mean, median, and min of ROI-specific values; (D) Estimated integrand surface for linear QIs based on Ki-67 CSI expression levels with estimated coefficient function β(p) (in purple) shown in the *qs*-plane; (E) Estimated integrand surface for nlQIs based on Ki-67 CSI expression levels. The contour lines on the integrand surfaces show the paths of integration for computing Ki-67 QIs for a subsample of tissue cores. Projections of these paths onto (p,q) plane show the corresponding Ki-67 CSI sample quantiles. The areas under the projections of the integrand paths onto (p,s) plane are equal to linear QI (D) or nlQIs (E).

Single-cell mIF-IHC imaging data are the result of digital processing of the microscopic images of tissue stained with selected antibodies. Figure 1A shows an example of an mIF-IHC image of a tissue core stained for Ki-67 protein as well as DAPI and cytokeratin. Quantitative pathology platforms, such as Akoya or QuPath, support cell segmentation of mIF-IHC images and quantification of the mean protein expression in each cell. The cell centroid coordinates and cell signal intensities (CSIs) for each stained protein are usually extracted as individual comma-separated values (.csv) files. It is expected that for each independent cluster (subject), a large number of observations are available, capturing information about each cell in the subject tissue. In some but not all cases, single-cell data would come from Regions of Interest (ROI) selected in the whole tissue image. Thus, the ROIs are clustered within subjects, and each ROI includes a large number of single-cell observations. Alternatively, a Tissue Microarray (TMA) may be analyzed with multiple tissue cores per subject. In either case, ROIs or TMA tissue cores represent the lowest level of clustering in the data with a substantially varying number of cells per cluster. Figure 1B illustrates the structure of the mIF-IHC CSI data with two levels of clustering and an outcome of interest (Response). The actual mIF-IHC data sets may include CSI levels for multiple proteins and possibly variables capturing phenotypes of each cell (e.g. cancer, T-cell, macrophage, etc.).

Prior to analysis, function as.groupedHyperframe() in the R packages groupedHyperframe is used to convert the CSI data into an R object of groupedHyperframe class. Data example Ki67 in package groupedHyperframe is a grouped hyper data frame, an extension of the hyper data frame defined in package spatstat.geom (Baddeley and Turner, 2005, Baddeley *et al.* 2015). The numeric-hypercolumn logKi67, whose elements are numeric vectors of different lengths, contains the log-transformed Ki-67 protein expression CSIs in each ∼patientID/tissueID, a nested grouping structure following the nomenclature of package nlme (Pinheiro et al. 2025). The data example Ki67 also contains the metadata, e.g. progression-free survival (PFS), Her2, HR, etc.

### Quantile index (QI) biomarkers

Linear quantile index (QI, Yi *et al.* 2023a) is a predictor in a functional generalized linear model (James, 2002) for outcomes from the exponential family of distributions, or a linear functional Cox model (Gellar *et al.* 2015) for survival outcomes,


(1) $${\text{QI}}_{i}=\int_{0}^{1}\beta (p)Q_{i}(p)dp,$$

where $Q_{i}(p)$ is the sample quantiles for the *i*-th patient and β(p) is an unknown coefficient function.

Non-linear quantile index (nlQI, Yi *et al.* 2025) is a predictor in a functional generalized additive model (McLean *et al.* 2014) for outcomes from the exponential family of distributions or an additive functional Cox model (Cui *et al.* 2021) for survival outcomes.


(2) $${\text{nlQI}}_{i}=\int_{0}^{1}F(p,Q_{i}(p))dp$$

where F(·,·) is an unknown bivariate twice differentiable function, which allows the association between the nonlinear quantile index and the functional predictor $Q_{i}(p)$ to vary nonlinearly in both the functional domain *p* and the value of the functional predictor $Q_{i}(p)$.

### Pipeline for deriving QI biomarkers

Complete R code for the analysis outlined in the following steps can be found in the vignette of package hyper.gam at https://CRAN.R-project.org/package=hyper.gam/vignettes/applications.html, in the section *Quantile Index*. The vignette covers all technical details, including the R version and key dependencies, in the section *Introduction*.

#### Step 1: Compute aggregated quantiles

Function aggregate_quantile() converts each element of the hypercolumn in the groupedHyperframe object into sample quantiles at a pre-specified grid of probabilities $\left\{p_{k},k=1,\ldots ,K\}\in [0,1\right]$. If only one level of clustering is present in the data (e.g. only one ROI or tissue core per patient), the cluster-specific sample quantile function is used as the functional predictor for each patient. In case of multiple lowest-level clusters per patient (e.g. multiple tissueID’s per patientID), the function aggregate_quantile() aggregates the lowest-level cluster-specific sample quantiles into patient-specific functions using point-wise mean, median, minimum and maximum (Fig. 1C). The aggregation must be performed at the level of biologically independent clusters, e.g. ∼patientID, to produce independent aggregated quantile predictors.

Figure 1D and E shows the (aggregated) patient-specific sample quantiles in the horizontal *pq*-plane with coordinates *p* (probability) and *q* (quantile).

#### Step 2: Estimate integrand surface

Function hyper_gam() estimates a scalar-on-function model with patient-specific aggregated sample quantile functions as a functional predictor. It returns an object of class hyper_gam, which inherits from the gam class of package mgcv (Wood, 2017).

The estimated integrand surface,


(3) $$\hat{S}(p,q)=\{\begin{array}{cc} \hat{\beta }(p)\cdot q & \text{for}\;\text{QI}\;(1)\\ \hat{F}(p,q) & \text{for}\;\text{nlQI}\;(2) \end{array}$$

is defined for p∈[0,1], $q\in \text{range}\{Q_{i}(p),i=1,\ldots ,n\}$.

We also implement an optional sign adjustment to ensure a *positive correlation* of quantile indices with $Q_{i}(\tilde{p})$ at a user-specified $\tilde{p}$ (default $\tilde{p}=.5$), in order to facilitate the interpretation of quantile indices. The sign-adjusted integrand surface is ${\hat{S}}^{*}(p,q)=\hat{c}\cdot \hat{S}(p,q)$, where $\hat{c}=\text{sign}\left(\text{corr}\left(Q_{i}(\tilde{p}),{\hat{\text{QI}}}_{i}\right)\right)$ or $\text{sign}\left(\text{corr}\left(Q_{i}(\tilde{p}),{\hat{\text{nlQI}}}_{i}\right)\right)$.

#### Step 3: Compute quantile index predictor

Function predict.hyper_gam() computes QI and nlQI predictors for a training or independent test data set, based on the estimated integrand surface (3). The resulting QIs or nlQIs can be evaluated as a predictor of outcome of interest in the test data. The QI and nlQI computed in the training data set are expected to be optimistically biased as predictors of outcome because they are optimized on the same data set.

#### Visualization of integrand surface

Function integrandSurface() creates an interactive htmlwidgets (Vaidyanathan *et al.* 2023) visualization of the estimated integrand surfaces for the linear (1) or nonlinear quantile index (2) using package plotly (Sievert, 2020).

Snapshots in Fig. 1D and 1E show the estimated integrand surfaces for linear and nonlinear quantile index, respectively, in the training data set. The estimated integrand paths per patient are shown on the integrand surfaces (3), as well as projected to the (p,s)-plane. In addition, the estimated functional coefficient $\hat{\beta }(p)$ is shown projected to (p,s)-plane as a solid line in Fig. 1D.

## Discussion

R packages groupedHyperframe and hyper.gam implement a supervised learning framework for deriving single-index biomarkers based on distribution quantiles. The framework was designed to enable derivation of functional protein biomarkers based on single-cell protein expression levels with optimal predictive value for an outcome of interest. In our examples, empirical quantile functions represent CSI levels in mIF-IHC data, but the same methods and implementation are also applicable to any single-cell data from genomics, transcriptomics, proteomics, or metabolomics studies with quantitative expression levels. Currently publicly available single-cell data do not include numbers of subjects sufficient for the proposed supervised learning and testing in an independent data set, but rapidly evolving technologies are expected to produce such large sample data in the nearest future. Thus, the proposed methodology has the potential to facilitate the development of various single-cell omics biomarkers critically needed for personalized medicine.
